# HupA, the main undecaprenyl pyrophosphate and phosphatidylglycerol phosphate phosphatase in *Helicobacter pylori* is essential for colonization of the stomach

**DOI:** 10.1371/journal.ppat.1007972

**Published:** 2019-09-05

**Authors:** Elise Gasiorowski, Rodolphe Auger, Xudong Tian, Samia Hicham, Chantal Ecobichon, Sophie Roure, Martin V. Douglass, M. Stephen Trent, Dominique Mengin-Lecreulx, Thierry Touzé, Ivo Gomperts Boneca

**Affiliations:** 1 Institut Pasteur, Unité biologie et génétique de la paroi bactérienne, 28, rue du Docteur Roux, Paris, France; 2 INSERM, Groupe Avenir, Paris, France; 3 Université Paris Descartes, Sorbonne Paris Cité, Paris, France; 4 Institute for Integrative Biology of the Cell (I2BC), CEA, CNRS, Univ Paris-Sud, Université Paris-Saclay, Gif-sur-Yvette, France; 5 Department of Infectious Diseases, College of Veterinary Medicine, University of Georgia, Georgia, United States of America; 6 Department of Microbiology, Franklin College of Arts and Sciences, University of Georgia, Georgia, United States of America; 7 Center for Vaccines and Immunology, University of Georgia, Georgia, United States of America; University of Alberta, CANADA

## Abstract

The biogenesis of bacterial cell-envelope polysaccharides requires the translocation, across the plasma membrane, of sugar sub-units that are produced inside the cytoplasm. To this end, the hydrophilic sugars are anchored to a lipid phosphate carrier (undecaprenyl phosphate (C_55_-P)), yielding membrane intermediates which are translocated to the outer face of the membrane. Finally, the glycan moiety is transferred to a nascent acceptor polymer, releasing the carrier in the “inactive” undecaprenyl pyrophosphate (C_55_-PP) form. Thus, C_55_-P is generated through the dephosphorylation of C_55_-PP, itself arising from either *de novo* synthesis or recycling. Two types of integral membrane C_55_-PP phosphatases were described: BacA enzymes and a sub-group of PAP2 enzymes (type 2 phosphatidic acid phosphatases). The human pathogen *Helicobacter pylori* does not contain BacA homologue but has four membrane PAP2 proteins: LpxE, LpxF, HP0350 and HP0851. Here, we report the physiological role of HP0851, renamed HupA, via multiple and complementary approaches ranging from a detailed biochemical characterization to the assessment of its effect on cell envelope metabolism and microbe-host interactions. HupA displays a dual function as being the main C_55_-PP pyrophosphatase (UppP) and phosphatidylglycerol phosphate phosphatase (PGPase). Although not essential *in vitro*, HupA was essential *in vivo* for stomach colonization. *In vitro*, the remaining UppP activity was carried out by LpxE in addition to its lipid A 1-phosphate phosphatase activity. Both HupA and LpxE have crucial roles in the biosynthesis of several cell wall polysaccharides and thus constitute potential targets for new therapeutic strategies.

## Author summary

*Helicobacter pylori* colonizes the human’s gastric mucosa and infects around 50% of the world’s population. This pathogen is responsible for chronic gastritis, peptic ulcers and in worst cases leads to gastric cancer. It has been classified as a class I carcinogen by the World Health Organization in 1994. Here, we show that HP0851, renamed HupA, is the major undecaprenyl pyrophosphate (C_55_-PP) phosphatase (UppP) and the major phosphatidylglycerol phosphate phosphatase (PGPase). This enzyme is also involved in cationic antimicrobial peptide (CAMP) resistance to which *H*. *pylori hupA* mutant shows an increased sensitivity (4 fold). This mutant was unable to colonize the stomach in mouse model of infection showing that even if *hupA* was not essential *in vitro*, this gene was essential *in vivo*. Both HupA and LpxE have crucial roles in the biosynthesis of several cell wall polysaccharides and thus constitute potential targets for new therapeutic strategies.

## Introduction

The biogenesis of many bacterial cell-envelope polysaccharides (i.e., peptidoglycan (PGN), lipopolysaccharides (LPS), teichoic acids, enterobacterial common antigen) requires the translocation, across the cytoplasmic membrane, of glycan units that are produced inside the cytoplasm [1]. Therefore, the hydrophilic sugars must be anchored to a lipid carrier (undecaprenyl phosphate (C_55_-P)), yielding membrane intermediates which are translocated to the outer face of the membrane [2]. In PGN biosynthesis, these intermediates are finally cross-linked by transglycosylase and transpeptidase activities to a nascent acceptor polymer. These polymerization reactions release the lipid carrier in an “inactive” undecaprenyl pyrophosphate form (C_55_-PP) which must be recycled to participate in new rounds of cell-envelope polysaccharides biosynthesis.

C_55_-P originates from the dephosphorylation of C_55_-PP, itself arising from either (*i*) cytoplasmic *de novo* synthesis by condensation of eight isopentenyl pyrophosphate (C_5_-PP) molecules with one farnesyl pyrophosphate (C_15_-PP) catalyzed by the essential C_55_-PP synthase (UppS) [3] or (*ii*) recycling [4] when it is released at the periplasmic side of the membrane. Two unrelated families of integral membrane proteins exhibiting C_55_-PP phosphatase (UppP) activity were identified: BacA and members of the PAP2 (type 2 phosphatidic acid phosphatase) super-family. *Escherichia coli* cells possess four UppPs: BacA enzyme which accounts for 75% of UppP activity and three PAP2 enzymes (PgpB, YbjG and LpxT) which ensure the remaining activity [5]. The plurality of UppPs as observed in *E*. *coli* and *Bacillus subtilis* [6] seems to be shared by most of the bacteria as suggested by a search for homologues, raising the question of the role of such a multiplicity. The simultaneous inactivation of *bacA*, *ybjG* and *pgpB* is lethal in *E*. *coli* whereas any single or double deletions had no effect on bacterial growth. Overexpression of BacA, PgpB or YbjG resulted in bacitracin resistance, and an increase of the UppP activity contained in membrane extracts [7]. Bacitracin is an antibiotic produced by *Bacillus licheniformis* [8] which strongly binds C_55_-PP, thereby inhibiting its dephosphorylation and leading to an arrest of PGN biosynthesis. When overexpressed, the UppP enzymes likely compete with the bacitracin for C_55_-PP binding, thus favoring its dephosphorylation. LpxT was not able to sustain growth of the triple *bacA-ybjG-pgpB* mutant and its overexpression did not lead to any bacitracin resistance suggesting that LpxT displays another function. Nevertheless, LpxT was shown to catalyze the transfer of C_55_-PP distal phosphate group onto lipid A, the lipid moiety of LPS, yielding C_55_-P and a pyrophosphorylated form of lipid A [9]. In addition to its UppP activity, PgpB is involved in phospholipids biosynthesis *via* the hydrolysis of phosphatidylglycerol phosphate (PGP) to form phosphatidylglycerol (PG) [10]. In *E*. *coli*, PgpA and PgpC are two additional integral membrane enzymes sharing the latter function. It has been shown that the co-inactivation of the three PGP phosphatases leads to a lethal phenotype [11]. Topology and structural analyses of PAP2 enzymes from *E*. *coli* and *B*. *subtilis* showed that these enzymes exhibit their active site at the interface between the plasma membrane and the periplasmic space, suggesting they are rather involved in C_55_-PP recycling [6,12,13].

More recently, the structure of BacA from *E*. *coli* was also resolved [14,15] showing here again an enzyme with its active site residues accessible from the periplasmic side. Nevertheless, the unique structure of BacA was reminiscent of that of transporters or channels raising the possibility that BacA may have alternate active sites on either side of the membrane and/or may function as a flippase allowing complete recycling of C_55_-P.

*Helicobacter pylori* is a microaerophilic, spiral-shaped, flagellated, Gram-negative bacterium that colonizes the human’s gastric mucosa [16]. This pathogen is responsible of chronic gastritis and peptic ulcers [17] and is a risk factor for gastric cancer. It has been classified as a class I carcinogen by the World Health Organization in 1994. In contrast to *E*. *coli*, *H*. *pylori* does not contain a BacA protein but has four PAP2 enzymes: HP0021 (LpxE), HP0350, HP0851 and HP1580 (LpxF). LpxE and LpxF have been shown to be involved in LPS modifications. The lipid A structure of *H*. *pylori* is unique and constitutively modified. LpxE is responsible for the removal of the lipid A 1-phosphate group that is required for the further addition of a phosphoethanolamine (PE) group at the same position [18]. LpxF is a lipid A 4’-phosphate phosphatase. Both modifications increase the net positive charge of the lipid A, which then confers cationic antimicrobial peptide (CAMP) resistance and escape to the host innate immune system, thereby allowing *H*. *pylori* to survive in the gastric mucosa [19]. The aim of this study was to decipher the physiological role of the multiple PAP2 enzymes from *H*. *pylori*. We describe that HP0851, renamed HupA, is the main UppP but is also involved in phospholipid biosynthesis by catalyzing the dephosphorylation of PGP in PG. In addition, we show that HupA has a role in CAMP resistance and is essential for colonization of the mouse stomach. In a global context of increasing resistance to antibiotics, finding new potential therapeutic targets is crucial especially against *H*. *pylori*, which colonizes half of the world’s population and is classified by the World Health Organization as a priority 2 pathogen regarding antibiotic resistance. A better understanding of essential metabolic pathways, and of the enzymes involved in such processes, could lead to the development of new antibacterials.

## Results

### Purification of PAP2 proteins and determination of their UppP activities

**Fig 1:**
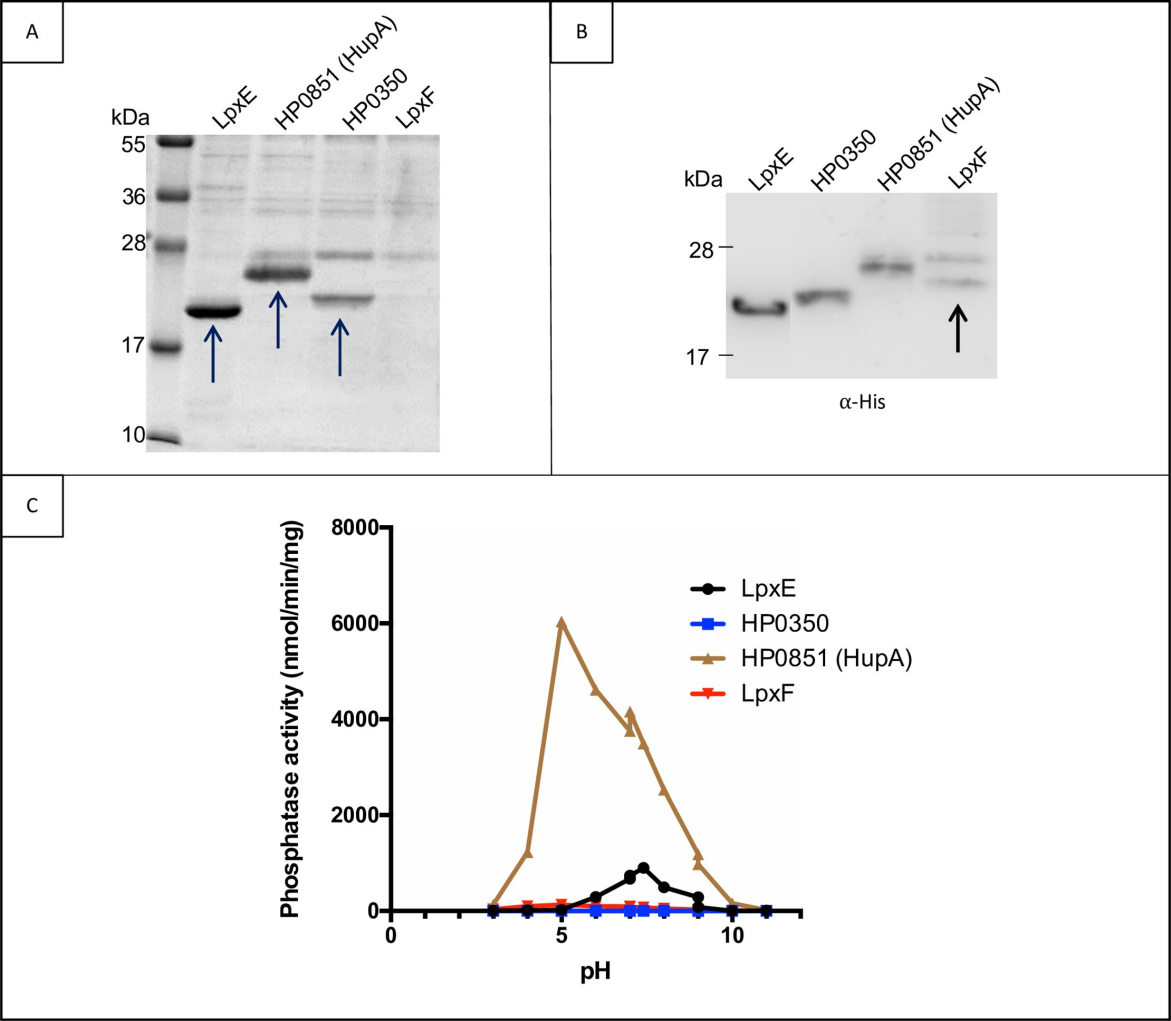
PAP2 proteins purification and effect of the pH on their UppP activity. The N-terminal His-tagged PAP2 proteins were purified on Ni^2+^-NTA-agarose beads as detailed in Materials and Methods, and 10 μL aliquots of the elution fractions were analyzed by SDS-PAGE. The proteins were revealed either (**A**) by Coomassie blue staining or (**B**) by Western blotting using anti·His tag antibody conjugated to the horseradish peroxidase (**C**) UppP phosphatase activity present in the purified solutions was determined at different pH (from pH 3 to pH 11). LpxE (black), HP0350 (blue), HP0851 (HupA; brown) and LpxF (red) The values are also reported in Table 2. The observed molecular weight of recombinant proteins are consistent with their calculated molecular weight: LpxE (21,2 kDa), HP0350 (24,5 kDa), HupA (25,8 kDa) and LpxF (24,6 kDa).

To characterize the PAP2 enzymes from *H*. *pylori* (LpxE, HP0350, HP0851 and LpxF), the corresponding genes were cloned on the p*Trc*His30 expression vector under the control of a strong IPTG-inducible promoter (**Table 1**). The recombinant N-terminally His-tagged proteins were overproduced in *E*. *coli* C43(*DE3*) cells. The integral membrane proteins were extracted from membranes via their solubilization with *n*-dodecyl-β-D-maltoside (DDM) detergent and the PAP2 proteins were purified by affinity chromatography using Ni^2+^-NTA agarose beads (Materials and Methods). Due to the difficulty in getting high expression levels of these membrane proteins in *E*. *coli*, they could not be purified to homogeneity. Nevertheless, LpxE, HP0350 and HP0851 were enriched to a high level as judged from SDS-PAGE analysis **(Fig 1A)**, while LpxF could not be visualized by Coomassie blue staining. However, LpxF was in the purified samples as confirmed by western blot analysis (**Fig 1B**). We then measured the UppP activities present in these purified solutions. Considering the niche of *H*. *pylori*, which ranges from the human stomach with an acidic pH to the epithelial interphase with a neutral pH, we measured enzymatic activities on a large range of pHs (**Fig 1C**).

**Table 1:** Bacterial strains and plasmids.

Strains	Genotype	Resistance^a^	Reference
***E*. *coli***			
DH5α	F^−^*endA1 glnV44 thi-1 recA1 relA1 gyrA96 deoR nupG purB20* φ80d*lacZ*ΔM15 Δ(*lacZYA-argF*)U169, hsdR17(*r*_*K*_^–^*m*_*K*_^+^), λ^–^		Life Science Technologies
C43(DE3)	F^−^*ompT gal dcm* hsdS_B_(*r*_*B*_^*-*^ *m*_*B*_^*-*^*)(DE3*)		Avidis
BWPGPTs	BW25113 Δ*pgpA* Δ*pgpC* Δ*pgpB*::Cm + pMAKkan*pgpB*	Cm, Kan	[6]
BWTs*bacA*	BW25113 Δ*bacA* Δ*ybjG* Δ*pgpB*::Kan + pMAK*bacA*	Cm, Kan	[7]
***H*. *pylori***			
HP-1	N6		[20]
HP-2	N6 *lpxE*::Gm	Genta	This work
HP-3	N6 *hp0350*::Km	Kan	This work
HP-4	N6 *lpxF*::Km	Kan	This work
HP-13	N6 *hupA*::Km	Kan	This work
HP-5	N6 pILL2150	Cm	This work
HP-7	N6 pILL2150 *bacA*	Cm	This work
HP-8	N6 pILL2150 *lpxT*	Cm	This work
HP-9	N6 pILL2150 *pgpB*	Cm	This work
HP-10	N6 pILL2150 *ybjG*	Cm	This work
HP-14	N6 pILL2150 *lpxE*	Cm	This work
HP-15	N6 pILL2150 *hp0350*	Cm	This work
HP-16	N6 pILL2150 *lpxF*	Cm	This work
N6 +pILL2157 *bacA*	N6 pILL2157 *bacA*	Cm	This work
N6 +pILL2157 *lpxT*	N6 pILL2157 *lpxT*	Cm	This work
N6 +pILL2157 *pgpB*	N6 pILL2157 *pgpB*	Cm	This work
N6 +pILL2157 *ybjG*	N6 pILL2157 *ybjG*	Cm	This work
HP-22	X47-2AL		[21]
HP-50	X47 *hupA*::Km	Kan	This work
HP-37	N6 *hupA*::Km pILL2150 *lpxE*	Kan, Cm	This work
HP-38	N6 *hupA*::Km pILL2150 *hp0350*	Kan, Cm	This work
HP-39	N6 *hupA*::Km pILL2150 *hupA*	Kan, Cm	This work
HP-40	N6 *hupA*::Km pILL2150 *lpxF*	Kan, Cm	This work
HP-33	N6 *hupA*::Km pILL2150	Kan, Cm	This work
HP-49	N6 *pgpA*::Km	Kan	This work
**Plasmids**			
Topo TA*ΔlpxE*:Gm		Genta	This work
Topo TA*ΔlpxE*:*Km*		Kan	This work
Topo TA*Δhp0350*:*Km*		Kan	This work
Topo TA*ΔhupA*:*Km*		Kan	This work
Topo TA*ΔlpxF*:*Km*		Kan	This work
p*Trc*His30 *lpxE*		Amp	This work
p*Trc*His30 *hp0350*		Amp	This work
p*Trc*His30 *hupA*		Amp	This work
p*Trc*His30 *lpxF*		Amp	This work
p*Trc*His30 *pgpA*		Amp	This work
pILL2150 *lpxE*		Cm	This work
pILL2150 *hp0350*		Cm	This work
pILL2150 *hupA*		Cm	This work
pILL2150 *lpxF*		Cm	This work
pILL2150 *bacA*		Cm	This work
pILL2150 *lpxT*		Cm	This work
pILL2150 *pgpB*		Cm	This work
pILL2150 *ybjG*		Cm	This work
pILL2157 *bacA*		Cm	This work
pILL2157 *lpxT*		Cm	This work
pILL2157 *pgpB*		Cm	This work
pILL2157 *ybjG*		Cm	This work

**(a)** Genta or Gm, Kan or Km, Cm and Amp: gentamycin, kanamycin, chloramphenicol and ampicillin resistance cassettes, respectively.

The uncharacterized HP0350 protein did not catalyze C_55_-PP dephosphorylation even under prolonged incubation time and high protein concentration. LpxE displayed an UppP activity of 900 nmol/min/mg at an optimal pH of 7.4, while HP0851 exhibited a 6.7-fold higher UppP activity of 6039 nmol/min/mg at an optimal pH of 5. LpxE displayed UppP activity *in vitro* in addition to its role as lipid A 1-phosphate phosphatase [19]. Considering the highest activity of HP0851, this protein may account for a large part of C_55_-P (re)generation *in vivo*. LpxF was shown to act as a lipid A 4’-phosphate phosphatase [19]. In this study, we also detected a low UppP activity for LpxF (**Table 2 and Fig 1C**). This activity is unlikely to arise from contaminants since HP0350, which was purified using the same methodology, exhibited no activity as compared to LpxF.

**Table 2:** UppP activity of *H*. *pylori* PAP2 enzymes.

	UppP specific activity (nmol/min/mg)			
pH	LpxE	HP0350	HP0851 (HupA)	LpxF
3	10	ND	131	30.8
4	13.8	ND	1227	97.2
**5**	**21.3**	**ND**	**6039**	**130.3**
6	291.3	ND	4616	97.5
7	671.3	ND	3755	91.8
7	735	ND	4157	84.8
7.4	900	ND	3493	71.5
8	492.5	ND	2528	48.8
9	285	ND	1180	28.7
9	76.3	ND	974	20.5
10	0	ND	159	9
11	8.8	ND	19	4.2

The enzymatic activity was measured in the presence of 50 μM of [^14^C]C_55_-PP substrate and an appropriate amount of enzyme to obtain less than 30% of hydrolysis. Buffering of the reaction mixture was obtained with sodium acetate (pH 3–7), Tris-HCl (pH 7–9) or sodium carbonate (pH 9–11). The C_55_-P product and the substrate were separated by TLC and further quantified by radioactivity counting. Values represent the mean of at least three individual experiments (the S.D. being within 15% of the presented values). ND, no detectable activity.

### HP0851 accounts for more than 90% of the UppP activity

**Fig 2:**
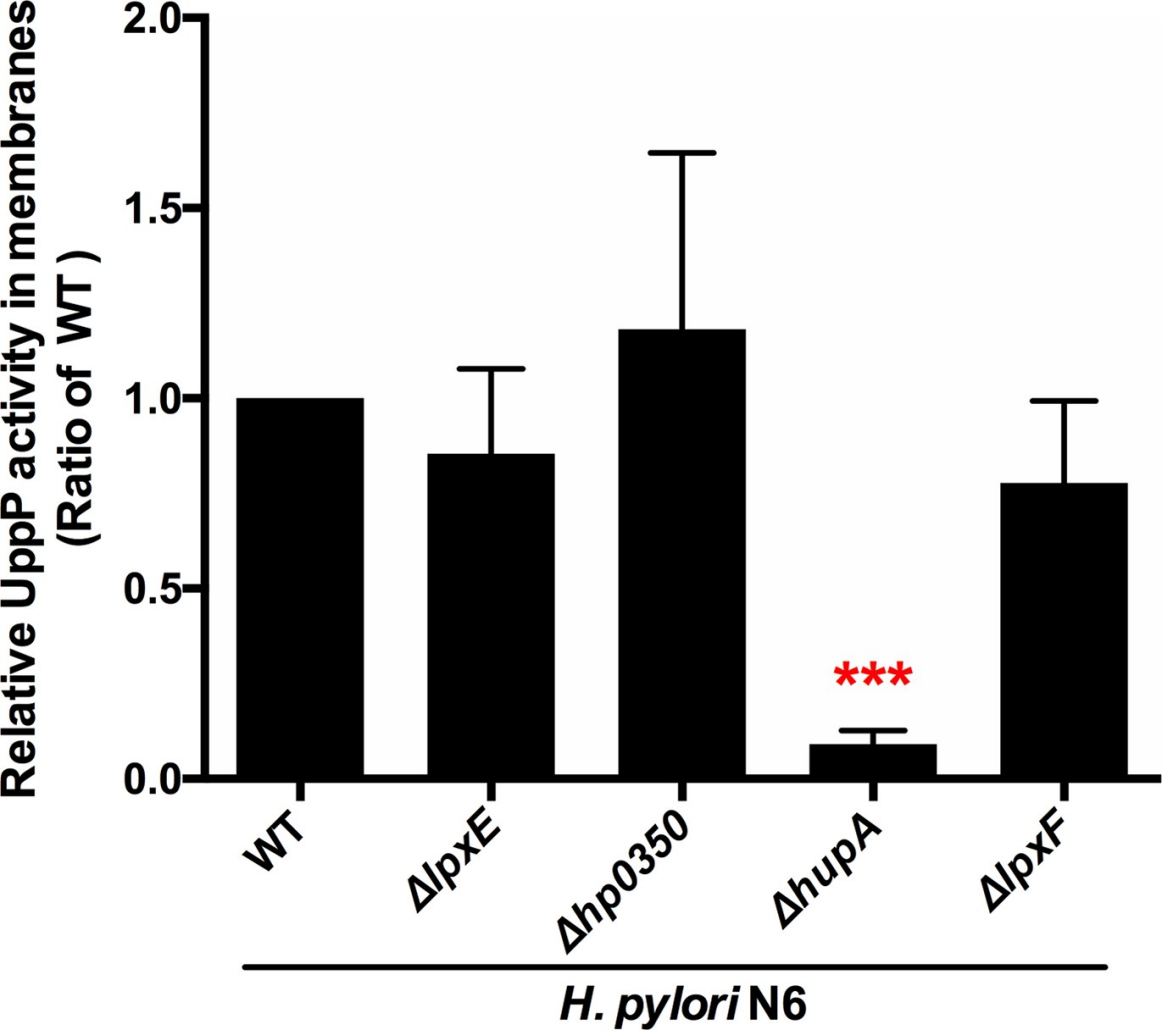
UppP activity in membranes of *H*. *pylori*. The UppP activity of the wild-type and the four single mutants of *H*. *pylori* N6 strain was measured and normalized by the quantity of proteins in each membrane extracts. The relative activities as compared to the wild-type strain membrane extracts are indicated. Each value is the mean of three independent measurements.

To further address the contribution of each PAP2 protein in the global UppP activity in *H*. *pylori*, we generated the four corresponding single mutants in *H*. *pylori* N6. All four mutants were readily obtained showing that none of these proteins is essential for survival of *H*. *pylori in vitro*. We prepared DDM-solubilized membrane extracts from exponentially growing wild-type and mutant cells and we measured the residual UppP activity present in these extracts (**Fig 2**). The UppP activities present in membranes of *hp0350*, *lpxE and lpxF* single mutants and wild-type strain were very similar. In contrast, the UppP activity decreased in *hp0851* membranes to about 10% of residual activity as compared to the wild-type. Thus, these data confirmed the major contribution of HP0851 in C_55_-PP recycling in C_55_-P.

### Only LpxE and HP0851 complement the *E*. *coli* conditionally UppP deficient strain

The simultaneous inactivation of *bacA*, *pgpB* and *ybjG* is lethal in *E*. *coli*. The BWTs*bacA* strain is a thermosensitive conditional triple mutant (*ΔbacA*, *ΔybjG*, *ΔpgpB)* containing an ectopic copy of *bacA* on a plasmid whose replication is impaired at 42°C [7]. This mutant accumulates soluble PGN precursors and lyses after a shift from 30°C to 42°C, due to the depletion of the pool of C_55_-P that arrests cell-wall synthesis. The ability of the PAP2 proteins from *H*. *pylori* to restore the growth of BWTs*bacA* at the restrictive temperature was tested using the p*Trc*His30-based plasmids previously used for protein purification (**Table 3**). The *hp0350* gene was unable to complement BWTs*bacA* strain even in the presence of 1 mM IPTG. The *lpxF* gene was also unable to complement in the absence of inducer and was found to be toxic in the presence of IPTG as no growth was observed either at 30°C or at 42°C. In contrast, *lpxE* and *hp0851* genes complemented BWTs*bacA* without the need of inducer, indicating that a basal level of the corresponding proteins allows a supply of C_55_-P that is appropriate for optimal growth of *E*. *coli*. The overproduction of LpxE and LpxF in *E*. *coli*, due to the addition of IPTG, was lethal possibly due: 1) to interference with LPS biosynthesis, since the modifications they catalyze do not normally exist in *E*. *coli* and likely generate cytotoxicity, and/or 2) to accumulation of large amounts of membrane proteins. These complementation assays perfectly corroborate the previous biochemical data, further demonstrating that LpxE and HP0851 act as the major and perhaps the sole C_55_-PP phosphatases in *H*. *pylori*. Hence, we renamed HP0851 to HupA (*H**elicobacter* UppP and PGPase A).

**Table 3:** Functional complementation of *E*. *coli* BWTs*bacA* conditional strain by PAP2 encoding genes from *H*. *pylori*.

*E*. *coli* BWTs*bacA*	CFU/mL			
	30°C	42°C	30°C +IPTG 1mM	42°C +IPTG 1mM
-	782	0	876	1
+p*Trc* His30 *lpxE*	229	222	0	0
+p*Trc* His30 *hp0350*	396	2	354	0
+p*Trc* His30 *hupA*	265	219	186	172
+p*Trc* His30 *lpxF*	180	0	0	0

The *E*. *coli* thermosensitive strain was transformed with the p*Trc*His30-based plasmids and aliquots were plated onto two ampicillin-containing 2YT agar plates incubated at either 30°C or 42°C. The CFU were counted after 24 h incubation.

### The simultaneous inactivation of *lpxE* and *hupA* is lethal

To address whether LpxE and HupA are the only C_55_-PP phosphatases in *H*. *pylori*, we attempted to construct a *lpxE*/*hupA* double mutant. However, we failed to generate this strain suggesting that co-inactivation of both genes is lethal. We then performed transformation efficiency assays to test the capacity of each PAP2 from *H*. *pylori* to complement this apparent lethality. We generated four strains deleted for *hupA* and carrying a copy of one PAP2 encoding gene under the control of an IPTG-inducible promoter on the pILL2150 vector. We then transformed these cells with the Topo TA*ΔlpxE* plasmid in order to replace *lpxE* by a gentamycin resistance cassette. The transformed *H*. *pylori* population was diluted and spread on normal (total number of bacteria) or selective (Δ*lpxE* recombinants) plates to measure the transformation efficiency expressed in cfu/μg of Topo TAΔ*lpxE* plasmid (**Table 4**). LpxF and HP0350 were unable to complement the *lpxE/hupA* double mutant as no recombinant was obtained even in the presence of IPTG. In contrast, LpxE and HupA complemented the double mutant in the presence of IPTG. These data excluded LpxF and HP0350 as *bona fide* UppP and confirm that only LpxE and HupA are major UppPs in *H*. *pylori*.

**Table 4:** Complementation of *lpxE*/*hupA* double mutant with an ectopic copy of *H*. *pylori* PAP2 encoding genes.

*Helicobacter pylori* N6 *hupA*::*Km*	Transformation rate (transformants/cfu/μg *TopoTA*Δ*lpxE*)	
	-ITPG	+IPTG (1mM)
+pILL2150 empty	0	0
**+pILL2150 *lpxE***	**0**	**1.44E-05**
+pILL2150 *hp0350*	0	0
**+pILL2150 *hupA***	**0**	**1.16E-05**
+pILL2150 *lpxF*	0	0

Quantification of *lpxE* gene inactivation in *ΔhupA* single mutant transformed with plasmids expressing one PAP2 from *H*. *pylori*. The transformation rates were measured in the presence and in the absence of IPTG.

### HupA is involved in resistance to CAMPs

*H*. *pylori* infects the stomach and persists in the mucosa during years due to the expression of multiple virulence factors. Persistence also requires a resistance towards CAMPs, which are secreted by the host [22]. To evaluate the involvement of PAP2 proteins in CAMPs resistance, we used polymyxin B as a surrogate for CAMPs [23]. The modifications generated by LpxE and LpxF through their lipid A 1- and 4’-phosphate phosphatase activities promote resistance to different CAMPs and they further confer host innate immune system evasion [19]. We confirmed the increased sensitivity already described in [19] for the *lpxE* and *lpxF* mutants (32- and 128-fold, respectively, **Table 5**). The *hp0350* mutant did not show any increase of polymyxin B sensitivity, while the sensitivity of *hupA* mutant increased by four-fold as compared to the wild-type strain. The polymyxin B sensitivity of *hupA* mutant was fully restored upon complementation with an ectopic copy of *hupA* gene.

**Table 5:** Minimal Inhibitory Concentrations (MIC) of polymyxin B of *H*. *pylori* strains.

*Helicobacter pylori* N6	Polymyxin B	Fold change *vs* WT strain
WT	2048	-
*lpxE*::*Gm*	64	32
*lpxF*::*Km*	16	128
*hp0350*::*Km*	2048	1
*hupA*::*Km*	512	4
*hupA*::*Km* + pILL2157 *hupA*	2048	1

MICs are reported as μg/ml and are the average of three experiments.

### HupA has no effect on lipid A structure

As already mentioned, LpxE and LpxF modify lipid A structure in *H*. *pylori*. We then investigated whether the four-fold decrease in polymyxin B resistance of *hupA* mutant was a consequence of an altered lipid A structure. LpxE exhibits UppP activity while also acting as a lipid A 1-phosphate phosphatase. We hypothesized that, in the absence of HupA, LpxE has to cope with this very low UppP activity. This hypothesis predicted the presence of a mixture of lipid A species in *hupA* mutant with lipid A 1-phosphate together with the usual lipid A 1-PE. The resulting rise in net negative charges at the outermost surface of the bacterium could then account for the increased sensitivity towards polymyxin B.

**Fig 3:**
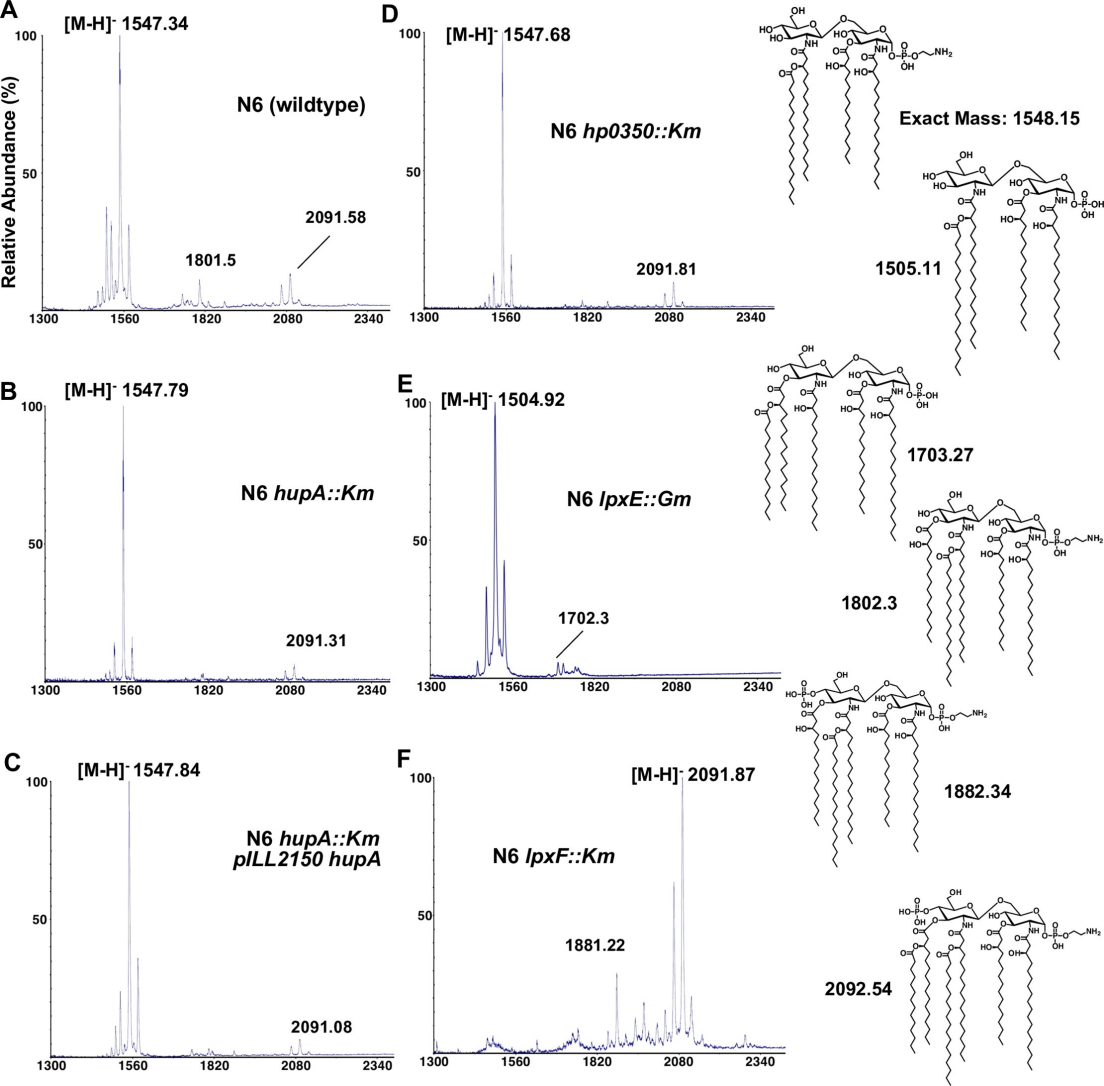
Mass spectrometry analysis of lipid A. Lipid A was isolated from wild-type (A), *hupA* (B), *hp0350* (D), *lpxE* (E) and lpxF (F) mutant and complemented cells (C) in strain N6 and analyzed by MALDI-TOF mass spectrometry in the negative-ion mode. The corresponding lipid A structure of the most abundant species is depicted for each analyzed strain.

To test this hypothesis, the lipid A from wild-type, *hupA* mutant and *hupA* mutant transformed with pILL2150 *hupA* plasmid were extracted and analyzed by mass spectrometry. As shown in **Fig 3A–3C**, the main form of lipid A present in wild-type strain was the tetra-acylated lipid A with a PE group at position 1 and no phosphate group at position 4’, as described [19]. Two additional minor species were observed, a penta-acylated lipid A with PE at position 1 and no phosphate at position 4’ and a hexa-acylated lipid A with PE at position 1 and with phosphate at position 4’. In *hupA* mutant and its complemented derivative, the lipid A profiles were very similar to that of the wild-type strain. We then concluded that the moderate decrease in polymyxin B resistance in *hupA* strain was independent of the lipid A structure. In parallel, we confirmed the roles of LpxE and LpxF (**Fig 3E and 3F**) while we show that HP0350 is not involved in lipid A modifications (**Fig 3D**).

### HupA is essential for stomach colonization

**Fig 4:**
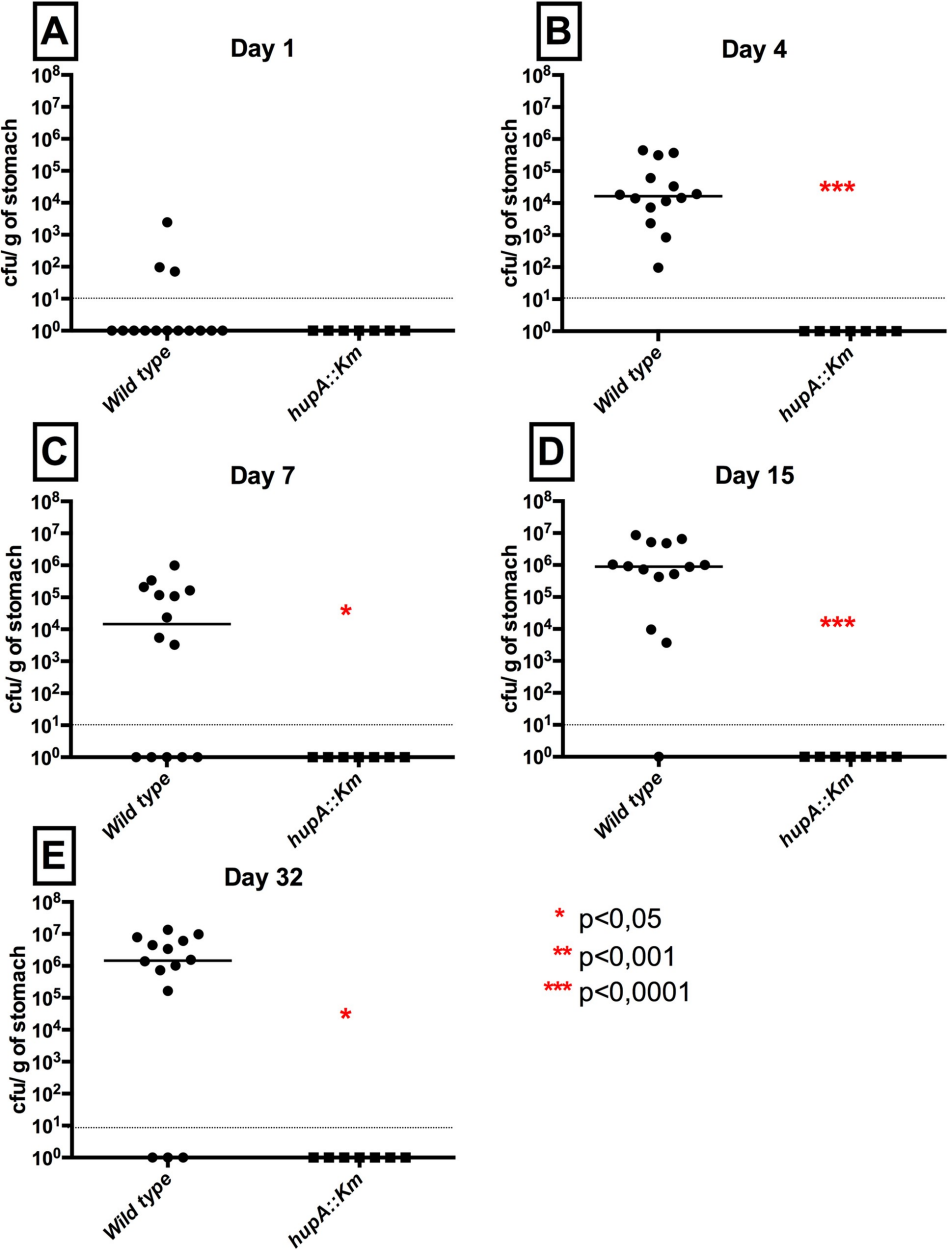
*In vivo* colonization of the *hp0851* mutant in strain X47 at days 1, 4, 7, 15 and 32 using OF1 mice. OF1 mice were infected orogastrically with the indicated strains at 2 × 10^8^ bacteria per mouse. Colonization rates were determined after 1 (**A**), 4 (**B**), 7 (**C**) 15 (**D**) and 32 (**E**) days by enumeration of colony forming units per gram (CFU/g) of stomach. Circles and square represent individual mice while mean colonization levels are illustrated by horizontal bars. Circles and square located on the x-axis represent mice with no colonization. The single *hp0851* mutant showed a statistically significant colonization defect at day 4, 7, 15 and 32 when compared to wild-type as indicated by a red asterisk (*p<0.05; ***p<0,001). Data from two independent cohorts of mice were combined to increase significance and robustness of our analysis.

**Fig 5:**
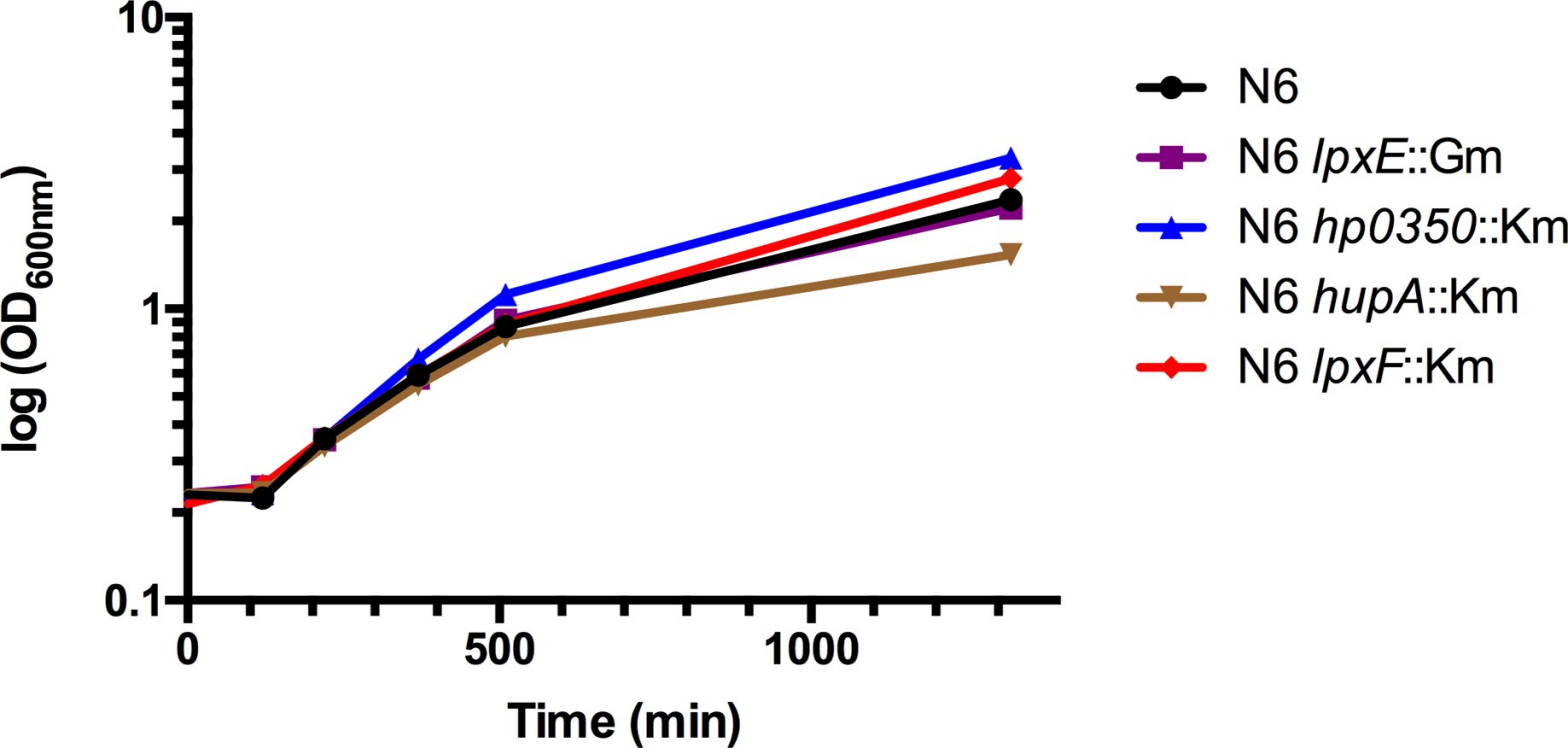
Growth curves of *Helicobacter pylori* N6. WT (black) and the four single mutants *lpxE*∷Km (purple), *hp0350*∷Km (blue), *hupA*∷Km (brown) and *lpxF*∷Km (red) were grown with classic conditions previously described in Bacterial strains, plasmids and bacterial growth conditions section.

The maximal UppP activity of LpxE was reached at pH 7.4 (**Fig 1**) and all complementation assays were performed at neutral pH. *H*. *pylori* resumes growth in acidic media only after buffering the environment to a pH above 6 thanks to the urease enzyme [24], which precludes any assays at acidic pH. However, in mouse colonization, the natural environment of *H*. *pylori* is mainly acidic, ranging from 2 in the lumen to 5–6 in the mucus layer. But at low pH, we noticed that the *in vitro* activity of LpxE drastically decreased by 43-fold (from 900 to 21 nmol/min/mg), while that of HupA increased by 1.7-fold (from 3493 to 6039 nmol/min/mg) (**Table 2)**. Therefore, LpxE may be unable to fully complement the reduced UppP activity in the *hupA* mutant in an acidic environment. This issue was then addressed by mouse colonization assays. The Δ*hupA* mutation was first introduced into the mouse-adapted strain *H pylori* X47 and the kinetics of colonization of wild-type and *hupA* strains was followed by sacrificing mice after 1, 4, 7, 15 and 32 days after bacterial inoculation. Seven mice per group were sacrificed in two independent experiments and **Fig 4** illustrates the CFU/g of stomach in a time course. The *hupA* mutant was unable to colonize the stomach neither at early or later stages after inoculation, showing therefore that HupA is absolutely essential for viability of *H*. *pylori in vivo*, in sharp contrast to *in vitro* where neither of the four PAP2 mutants was affected in growth (Fig 5).

### HupA has major involvement in phospholipid biosynthesis

HupA is an orthologue of PgpB from *E*. *coli*, which is a bifunctional enzyme acting as a minor UppP and being largely involved in phospholipid biosynthesis through the dephosphorylation of PGP in PG. In *E*. *coli*, the latter activity is also provided by two other integral membrane proteins named PgpA and PgpC, which are unrelated to the PAP2 superfamily [10,11,25]. One putative gene encoding a PgpA homolog was identified in the genome of *H*. *pylori*, *hp0737*, while no PgpC encoding gene was found. The *hp0737* was cloned on a p*Trc*His30 vector and the N-terminal-His tagged protein was expressed in *E*. *coli* C43(*DE3*) cells and purified from membranes to high homogeneity in DDM micelles (S1A Fig). In contrast to the PAP2 enzymes, PgpA enzymes are Mg^2+^-dependent, which was confirmed for HP0737 that presented an optimal Mg^2+^ concentration of 6 mM (S1B Fig). Its PGP phosphatase activity was then fully confirmed *in vitro* with a specific activity of 1138 ± 174 nmol/min/mg. Noticeably, HP0737 did not display any significant UppP activity and the plasmid carrying *hp0737* gene did not restore the growth of *E*. *coli* BWTs*bacA* strain at 42°C. Considering the activity of HP0737 both *in vivo* and *in vitro*, this protein was renamed PgpA. The *pgpA* null mutant was readily generated by replacement with a resistance cassette (**Table 1**). These disagree with a previous study in which *pgpA* was described as an essential gene [26]. Our data further suggested the existence of one or several other PGP phosphatases.

**Fig 6:**
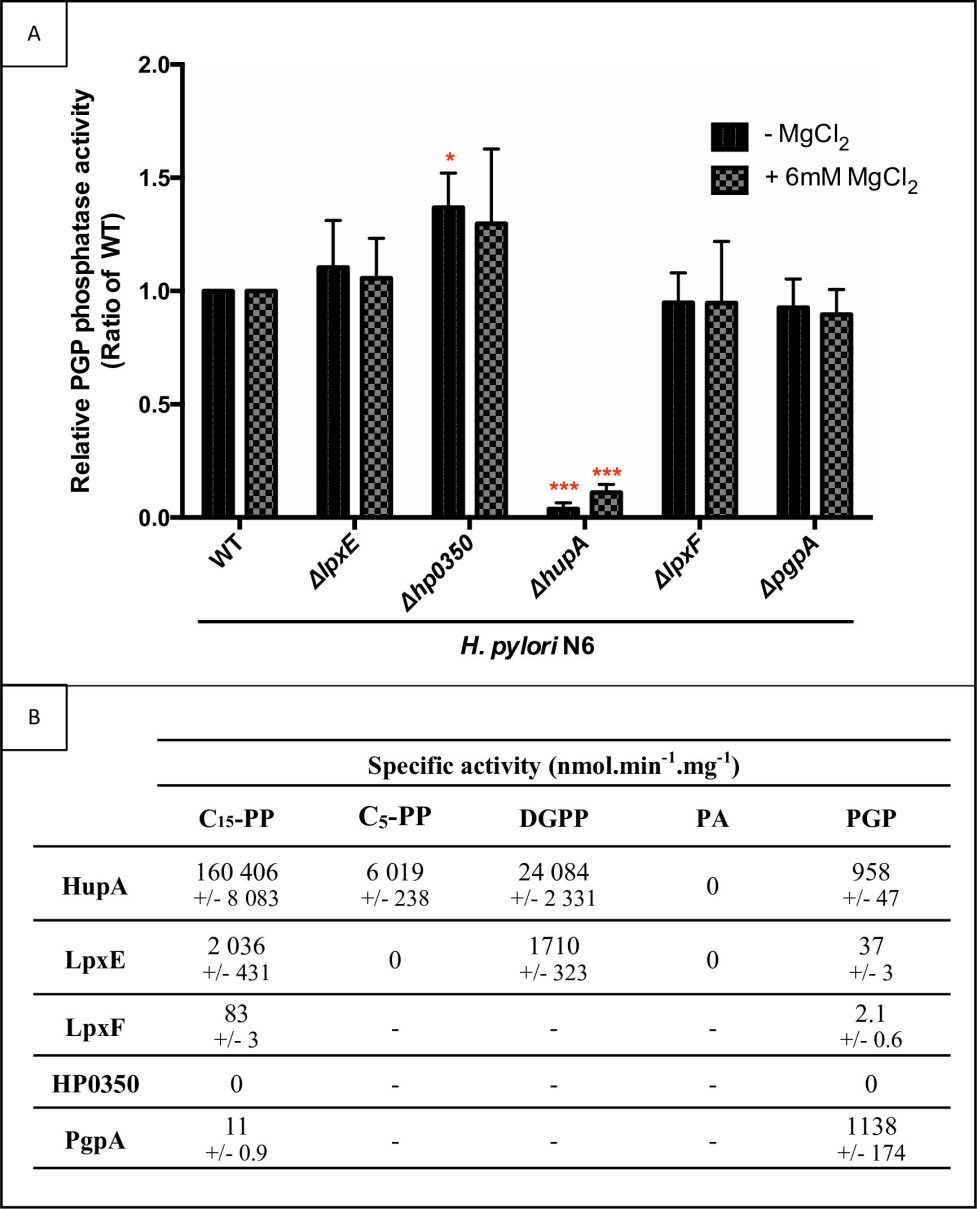
PGP phosphatase activities in membranes of *H*. *pylori*. The PGP phosphatase activity in presence or absence of MgCl_2_ of the wild-type and five single mutants of *H*. *pylori* N6 strain was measured and normalized by the quantity of proteins in membrane extracts (**A**). Ratios were then normalized by the wild-type strain. (B) The phosphatase specific activity of the recombinant proteins towards various substrates.

We then assessed whether the PAP2 proteins from *H*. *pylori* are also involved in the synthesis of PG. DDM-solubilized membrane extracts obtained from wild-type and mutant cells (*lpxE*, *hp0350*, *hupA*, *lpxF and pgpA*) were assayed for their PGP phosphatase activity. This activity was measured in the absence and presence of 6 mM Mg^2+^ (**Fig 6A**). A drastic decrease of the PGP phosphatase activity in the *hupA* mutant as compared to the wild-type strain was observed, while the other mutants displayed similar activities as the control. Under these conditions, HupA accounted for 98% of the PGP phosphatase activity in the membrane of *H*. *pylori*.

To further confirm the involvement of HupA in the biosynthesis of PG, we also performed complementation assays using the conditional BWPGPTs *E*. *coli* strain and the different p*Trc*His30-based plasmids. Like the BWTs*bacA* strain, the BWPGPTs is a thermosensitive triple mutant (*ΔpgpA*, *ΔpgpB*, *ΔpgpC)* containing an ectopic copy of *pgpB* on a plasmid whose replication is impaired at 42°C. LpxE, HupA and PgpA fully restored the growth at 42°C of this thermosensitive strain without IPTG, while LpxF and HP0350 were unable to complement (**Table 6**). Thus, LpxE, HupA and PgpA carry enough of PGP phosphatase activity to support growth of the BWPGPTs strain.

The apparent PGP phosphatase activity of HupA and LpxE *in vivo* could explain why *pgpA* inactivation did not lead to a lethal phenotype. To further decipher the role of PAP2 enzymes in the biosynthesis of PG in *H*. *pylori*, we tried to generate mutant strains inactivated for *hupA* and *pgpA* but we failed, while a double mutant *lpxE*/*pgpA* was readily obtained. These results suggest that HupA and PgpA are the only PGP phosphatases able to sustain growth of *H*. *pylori in vitro*.

**Table 6:** Complementation of *E*. *coli* BW*PGP*Ts conditional strain by PAP2 and PgpA encoding genes from *H*. *pylori*.

*E*. *coli* BW*PGP*Ts	CFU/mL	
	30°C	42°C
-	211	1
+p*Trc* His30 *lpxE*	97	140
+p*Trc* His30 *hp0350*	152	0
+p*Trc* His30 *hupA*	289	279
+p*Trc* His30 *lpxF*	75	0
+p*Trc* His30 *pgpA*	652	844

The *E*. *coli* thermosensitive strain was transformed with the p*Trc*His30-based plasmids and aliquots were plated onto two ampicillin-containing 2YT agar plates incubated at either 30°C or 42°C. The CFU were counted after 24 h incubation.

### Broad substrate specificity of *H*. *pylori* PAP2 enzymes

Since the HupA and LpxE accept very dissimilar lipidic substrates *in vivo*, we further analyzed the substrate specificity of PAP2 enzymes *in vitro* **(Fig 6B)**. Again, HP0350 had no visible phosphatase activity on any of the tested substrates. HupA was the most active phosphatase using all substrates tested except phosphatidic acid and the highest activity was by far obtained with the C_15_-PP substrate. Structural and topology analyses of integral membrane PAP2 enzymes revealed that their active site residues are oriented towards the periplasm, which is likely the same for HupA. Small molecules such as C_15_-PP are never exposed at the periplasmic site, therefore the C_15_-PP should not be a natural substrate *in vivo*. Nevertheless, this analysis demonstrates that HupA accepts a large range of pyro- and monophosphate substrates, in particular C_55_-PP and PGP. Noticeably, HupA and PgpA displayed very similar activities towards PGP. LpxE had similar substrate specificity as HupA, except for C_5_-PP, and much lower activities were found. In contrast, LpxF only showed very low activity towards C_15_-PP and PGP.

Since HupA showed broad substrate specificity, we wondered whether the polymyxin B sensitivity could be related to a general membrane permeability defect of the *hupA* mutant. We measured the MIC of the wild-type strain and its four PAP2 mutants to four different antimicrobial, one clinically relevant CAMP, colistin, and three large antibiotics, vancomycin, teicoplanin and daptomycin that do not cross the outer membrane. Table 7 illustrates the MICs to the four antimicrobials.

**Table 7:** Minimal Inhibitory Concentrations (MIC) of *H*. *pylori* strains.

*Helicobacter pylori* N6	Colistin	Vancomycin	Teicoplanin	Daptomycin
WT	R	R	R	R
*lpxE*::*Gm*	**12**	R	R	R
*lpxF*::*Km*	**1**	R	R	R
*hp0350*::*Km*	R	R	R	R
*hupA*::*Km*	**64**	R	R	R

MICs are reported as μg/ml or R: Resistant. R indicates above the highest concentration on the E-test, i.e. MIC >256 μg/mL.

Absence of HupA, LpxE and LpxF affected specifically resistance to CAMPs without affecting the resistance to large antimicrobials suggesting that these three PAP2 only affect overall membrane charge and not permeability. Given the specific effect on CAMPs resistance and the broad substrate specificity of HupA, we next analyzed the phospholipid composition of the *hupA* mutant. As illustrated in S2 Fig, the wild-type N6 and its four PAP2 mutants showed a similar phospholipid profile.

### PgpB, YbjG and BacA from *E*. *coli* are functional in *H*. *pylori*

Since, HupA and LpxE are functional in *E*. *coli*, we tested if the PAP2/BacA enzymes from *E*. *coli* are also functional in *H*. *pylori*. We performed complementation assays as previously described by measuring the transformation rate of *lpxE* inactivation in *hupA* mutant containing different plasmids encoding PAP2/BacA from *E*. *coli*. We then generated eight strains deleted for *hupA* and carrying a plasmid (pILL2150 or pILL2157 vector) bearing one of the four *E*. *coli* genes (*bacA*, *pbpB*, *ybjG* and *lpxT*) placed under the control of an IPTG-inducible promoter. The transgene is under control of a promoter from *H*. *pylori* in pILL2157, while it is under control of an *E*. *coli* promoter in pILL2150. Consequently, the transgenes in pILL2157 are likely more expressed in *H*. *pylori* as compared to those in pILL2150. Moreover, the pILL2157 promoter was previously found to be leaky in contrast to that of pILL2150 vector [27]. The transformation rates are summarized in **Table 8**. As expected, LpxT was unable to complement the lack of UppP activity in *H*. *pylori* whatever the expression level. At a moderate expression level (i.e. in pILL2150), only PgpB was able to complement the double *lpxE/hupA* mutant (1.08 × 10^−5^ transformants/cfu/μg DNA *topoTAΔlpxE*) in the presence of IPTG. In contrast, at a higher level of expression (i.e. in pILL2157), BacA, PgpB and YbjG were able to complement the double mutant both in the presence and in the absence of inducer. Therefore, even though BacA does not belong to the PAP2 super-family, and *H*. *pylori* does not possess UppP of the BacA type, this enzyme was functional in *H*. *pylori*.

**Table 8:** Complementation of *lpxE*/*hupA* double mutant with an ectopic copy of *E*. *coli* PAP2/BacA encoding genes.

*Helicobacter pylori* N6 *hupA*::*Km*	Transformation rate (transformants/cfu/μg *TopoTA*Δ*lpxE*)	
	-ITPG	+IPTG (1mM)
+pILL2150 empty	0	0
+pILL2150 *bacA*	0	0
+pILL2150 *lpxT*	0	0
**+pILL2150 *pgpB***	**0**	**1.08E-05**
+pILL2150 *ybjG*	0	0
**+pILL2157 *bacA***	**3.85E-05**	**5.25E-05**
+pILL2157 *lpxT*		
**+pILL2157 *pgpB***	**1.51E-05**	**2.07E-05**
**+pILL21570 *ybjG***	**5.86E-06**	**1.06E-06**

Quantification of *lpxE* gene inactivation in *ΔhupA* single mutant transformed with plasmids expressing one PAP2/BacA from *E*. *coli*. Two types of expression vectors were used: pILL2150 and pILL2157. The pILL2150 vector possesses an *E*. *coli* promoter and leads to low levels of expression in *H*. *pylori*, while pILL2157 displays a *H*. *pylori* promoter yielding high levels of expression in *H*. *pylori*. The transformation rates were measured in the presence and in the absence of IPTG.

## Discussion

The recycling of C_55_-PP is an essential step for the biosynthesis of many polysaccharides such as PGN, LPS O-antigen or teichoic acid [4]. This pathway was only studied in the Gram-positive bacterium *B*. *subtilis* [6] and more intensively in the Gram-negative *E*. *coli*. Two enzymes from *H*. *pylori* belonging to the PAP2 super-family, LpxE and LpxF, were previously demonstrated to have critical functions through the dephosphorylation of the lipid A moiety from LPS. The other two members of the PAP2 super-family, HupA and HP0350, were not characterized. HupA and HP0350 are orthologues of PgpB from *E*. *coli*, which was described as a dual functional enzyme exhibiting both C_55_-PP and PGP phosphatase activities, thus involved in both cell-envelope polysaccharides and phospholipids biosynthesis [11].

Here, we demonstrated, by *in vitro* and *in vivo* analyses, that HupA constitutes the major C_55_-PP phosphatase in *H*. *pylori*, being responsible for 90% of the total UppP activity present in the membranes. Interestingly, HupA presented an optimal pH of 5 for its enzymatic activity, which is close to the pH that *H*. *pylori* cells face in the human stomach. To further confirm this function, we showed that HupA was also fully functional in *E*. *coli* by complementing a PAP2/BacA conditionally deficient strain. The deletion of *hupA* gene was not lethal suggesting that one or several other C_55_-PP phosphatases exist in *H*. *pylori*. Among the three other PAP2, we showed that LpxE and LpxF exhibited UppP activity with an optimal pH of 7.4 and 5, respectively. However, only LpxE was also capable of complementing the conditional *E*. *coli* strain. The inactivation of both *lpxE* and *hupA* genes was found to be lethal, which was further confirmed by complementation assays of the double mutant by ectopic copies of *lpxE* or *hupA*. All these data confirmed that HupA and LpxE are the only physiologically relevant UppP in *H*. *pylori*.

In addition, we demonstrated that HupA plays critical roles *in vivo*. Indeed, HupA appeared important for the resistance towards CAMPs by a yet uncharacterized process. In this study, we excluded potential alteration of the structure of lipid A. More importantly, HupA was shown to be essential for stomach colonization. Indeed, the PAP2 super-family members all have their active site exposed to the periplasmic space where the pH is determined by the environmental pH. We then hypothesized, based on *in vitro* data, that LpxE enzyme might not be sufficiently active at acidic pH to sustain growth in the absence of HupA. Indeed, during colonization, LpxE is unlikely to function properly since it was poorly active at pH 5 *in vitro*. In fact, apart from the niche that is in contact with the epithelial cells where there exists a neutral pH, *H*. *pylori* cells are always exposed to an acidic environment. In these conditions, LpxE will likely be unable to provide the UppP activity required in absence of HupA enzyme.

The first step for stomach colonization by *H*. *pylori* is dependent on its motility and its urease enzyme to buffer its cytoplasm [24]. *H*. *pylori* escapes the lumen towards the mucus layer to reach a more favorable environment with a higher pH. Therefore, we cannot exclude a relevant physiological role of LpxE in C_55_-PP dephosphorylation after the first step of colonization, once *H*. *pylori* has reached the mucus layer and the epithelial surface. Our previous work on LpxE showed that LpxE is needed for long-term colonization. However, this is likely due to its role as lipid A 1-phosphate phosphatase and escape to TLR4 signaling rather than to its UppP activity since the mutant was able to colonize TLR4 KO mice [19].

This study also highlighted the dual function of HupA, which has also a major involvement in the biosynthesis of phospholipids through the synthesis of PG from PGP. By sequence homology, only one additional putative PGP phosphatase was found in *H*. *pylori*, i.e. HP0737, which is homologous to *E*. *coli* PgpA. A global transposon analysis performed on *H*. *pylori* reported *hp0737* as an essential gene [26]. However, we were able to delete *hp0737* gene by resistance cassette replacement. HP0737 could then account for the residual PGP phosphatase activity in *H*. *pylori hupA* null mutant. In *E*. *coli*, three PGP phosphatases were described, PgpA, PgpB and PgpC and only a triple mutant is lethal [11]. Even if the composition of phospholipids differs between *E*. *coli* and *H*. *pylori*, i.e. PG represents 25% and 12.5% of the total phospholipids, respectively [28,29], we can expect that the presence of PG is also essential in *H*. *pylori*, especially because PG is the precursor of another important phospholipid, the cardiolipin. A *pgpA*/*hupA* double mutant is lethal and confirms the importance of PG in *H*. *pylori*. Members of the PgpA-family have a predicted cytoplasmic active site while PAP2-like enzymes have a periplasmic oriented active site. These results suggest that PGP is accessible to dephosphorylation on both sides of the cytoplasmic membrane similarly to *E*. *coli*. Thus, HupA and PgpA are the only two physiologically relevant PGP phosphatases in *H*. *pylori* despite the weak PGP phosphatase activity of LpxE.

The involvement of HupA in phospholipid biosynthesis may explain the decrease of resistance to polymyxin B and colistin of the corresponding mutant. Indeed, these cationic peptides form pores in the cytoplasmic membrane leading to cytoplasmic leakage. Therefore, if the composition of the plasma membrane differs in the *hupA* mutant as compared to the wild-type strain, particularly with a possible accumulation of PGP, the membrane net negative charge might increase, favoring the binding of CAMPs, which could potentiate their capacity to insert within the membrane. However, our analysis of phospholipid composition of the wild-type N6 and its four PAP2 mutants showed similar phospholipid composition. Although TLC analysis is mainly qualitative, these results suggest that changes in membrane charge are unlikely to be due to an accumulation of PGP and suggest that accumulation of C55-PP might contribute to the mild increased sensitivity of the *hupA* mutant to CAMPs.

LpxE and HupA have similar broad substrate specificities although LpxE exhibits a much weaker activity. In contrast, LpxF has a very narrow substrate specificity and, so far, only catalyzes the dephosphorylation of the lipid A. It would be interesting to study the molecular basis of such distinct substrate specificity. The low sequence conservation among the membrane PAP2 proteins precludes *in silico* modelling studies using PgpB and *Bs*PgpB. Insights into substrate specificity will require 3D structures of HupA, LpxE and LpxF with their substrates.

In this report, we described HupA as the main C_55_-PP and PGP phosphatase in *H*. *pylori*. This protein was also essential for colonization, likely due to its dual functionality and its central role in several lipid biosynthetic pathways, suggesting that HupA is the sole enzyme capable to support C_55_-PP recycling and PG synthesis in its natural niche. Hence, HupA, as well as LpxE and LpxF, constitute *bona fide* new targets for innovative therapeutic strategies against *H*. *pylori*, which is becoming increasingly resistant to the existing antibiotic arsenal.

## Materials and Methods

### Ethics Statement

Animal experiments were done according to European (Directive 2010/63 EU) and French regulation (Décret 2013–118) under the authorized protocol CETEA 2014–072 reviewed by the Institut Pasteur Ethical Committee (registered as number 89 with the French Ministry of Research). The experimental protocol was also approved by the French Ministry of Research under the number APAFIS#11694–2017100510327765 v2.

### Bacterial strains, plasmids and bacterial growth conditions

The bacterial strains and plasmids used in the study are summarized in **Table 1.** Precultures of *H*. *pylori* were started from glycerol stocks routinely stored at -80°C, plated onto 10% horse blood agar medium supplemented with an antibiotic-antifungal mix [19] and incubated at 37°C for 24 h in a microaerobic atmosphere (6% O_2_, 10% CO_2_, 84% N_2_). The glycerol storage media is composed of 25% glycerol, 38% Brain Heart Infusion liquid (BHI) (Oxoid) and 37% sterilized water. Amplification of the preculture was performed in the same conditions as precultures in new plates at 37°C for 24 h in a microaerobic atmosphere. Liquid precultures were started from amplification plates and inoculated into BHI (Oxoid) supplemented with 10% fetal bovine serum (FBS) incubated at 37°C overnight in a microaerobic atmosphere. Liquid cultures of *H*. *pylori* were started from overnight liquid culture and inoculated into BHI (Oxoid) supplemented with 10% fetal bovine serum. In general, liquid cultures of *H*. *pylori* were grown to an OD_600nm_ of ~1.0 at 37°C under microaerobic conditions with shaking. Otherwise indicated, *E*. *coli* was routinely grown at 37°C in 2YT broth using standard conditions.

A non-polar kanamycin cassette was digested out of plasmid pUC18-Km2 [30] using BamHI and KpnI and ligated into the PCR products of the 5’and 3’ flanking regions of genes *lpxE*, *hp0350*, *hupA*, *lpxF and pgpA* using standard cloning techniques. The oligonucleotides used for the PCR amplification are described in S1 Table. The generated plasmids, TopoTA Δ*lpxE*:kan, TopoTA Δ*hp0350*:kan, TopoTA Δ*hupA*:kan, TopoTA Δ*lpxF*:kan and TopoTA Δ*pgpA*:kan were used to create the corresponding null mutants in *H*. *pylori* N6 and/or X47 by natural transformation. Similarly, a non-polar gentamycin cassette was digested out of plasmid pUC18-Gm [31] using BamHI and KpnI to generate TopoTA Δ*lpxE*:Gm.

For the expression of PAP2, BacA and PgpA encoding genes from *E*. *coli* or *H*. *pylori*, the genes were amplified by PCR using appropriate primers (S1 Table) and cloned into the pILL2150 or pILL2157 vectors (for expression in *H*. *pylori*) or in p*Trc*His30 vector (for expression in *E*. *coli*).

### Expression and purification of the PAP2 and PgpA proteins from *H*. *pylori*

*E*. *coli* C43(*DE3*) cells carrying p*Trc*His30-based plasmids were used for the overproduction of N-terminal His_6_-tagged proteins. Cells were grown in 1 liter 2YT-ampicillin at 37°C until the A_600nm_ reached 0.9, when the expression was induced by the addition of 1 mM IPTG and the growth was continued for 3 h at 37°C. Cells were harvested by centrifugation at 4°C for 20 min at 4000 × *g*, were washed and finally resuspended in buffer A (20 mM Tris-HCl pH 7.4, 400 mM NaCl, 10% glycerol) before disruption by sonication with a Vibracell 72412 sonicator (Bioblock). The suspension was centrifuged at 4°C for 1 h at 100,000 × *g* to harvest the membranes, which were washed three times in buffer A. The membranes were finally resuspended in buffer A supplemented with 2% DDM for solubilization at 4°C for 2 h with gentle agitation. Solubilized membranes were loaded on nickel-nitrilotriacetate agarose (Ni^2+^-NTA-agarose, Qiagen) equilibrated in buffer A supplemented with 0.2% DDM and 10 mM imidazole. The column was washed with increasing imidazole concentration using the same buffer and the elution was performed with 2 ml of buffer A supplemented with 0.2% DDM and 200 mM imidazole. Desalting of samples was carried out using PD-10 desalting columns (GE Healthcare) and buffer A supplemented with 0.1% DDM. Protein concentrations were determined with the QuantiProBCA assay kit (Sigma) or by densitometry analysis of the gel when appropriate.

### *H*. *pylori* membrane extracts preparation

*H*. *pylori* wild-type and single mutant strains were grown at 37°C in BHI medium (200 ml culture) up to exponential phase. Cell free extracts, membrane free cytosol, and washed membranes were prepared as previously described [22]. Cells were harvested by centrifugation, washed and finally resuspended in 20 mM Tris-HCl, pH 7.4, 200 mM NaCl (buffer B). After disruption by sonication, membranes were pelleted by centrifugation at 177 420 × g and resuspended in buffer B supplemented with 2% DDM for solubilization during 1.5 h in the cold. The solubilized proteins were recovered by centrifugation at 177 420 × g and conserved at -20°C before activity measurement.

### *H*. *pylori* membrane phospholipids extraction and staining

*H*. *pylori* wild-type and four single mutant strains were grown at 37°C in BHI medium (50 ml culture) up to exponential phase. Lipid extraction was performed using a new protocol (Nozeret K *et al*. manuscript submitted). For thin-layer chromatography (TLC) analysis of phospholipids, the dried lipid extracts and controls were dissolved respectively in 500μl and 350μl of chloroform. 10μl of the solution was spotted onto a TLC silica gel 60 plate. The TLC plate was developed in tanks equilibrated with dichloromethane- methanol-water (65:28:4 [vol/vol]). After drying the plate, phospholipids were visualized with molybdenum blue reagent (Sigma).

### C_55_-PP and PGP phosphatase assays

The C_55_-PP and PGP phosphatase assays were carried out in a 10 μl reaction mixture containing 20 mM Tris-HCl, pH 7.4, 10 mM β-mercaptoethanol, 150 mM NaCl, 0.2% DDM, 50 μM [^14^C]C_55_-PP or 50 μM [^14^C]PGP (900 Bq) and enzyme. MgCl_2_ was added at 6 mM in the reaction mixture when PgpA activity was measured. Appropriate dilutions of purified phosphatases, or of membrane extracts, were used to achieve less than 30% substrate hydrolysis. The reaction mixture was incubated at 37°C for 10 to 30 min and the reaction was stopped by freezing in liquid nitrogen. The substrates and products were then separated and quantified by thin layer chromatography (TLC) analysis, as previously described for C_55_-PP [5] and PGP [11] hydrolysis. When the phosphatase activity was investigated at various pH values, buffering was achieved in sodium acetate (pH 3–7), Tris-HCl (pH 7–9) or sodium carbonate buffer (pH 9–11).

The phosphatase activity towards other non-radiolabeled substrates: C_5_-PP, C_15_-PP, diacyl(C8) glycerol-PP (DGPP) and phosphatidic acid (PA), was determined by measuring the amount of released inorganic phosphate during catalysis. The reaction mixture was as described above with 50 μM substrate in a final volume of 50 μl. After 10 min of incubation at 37°C, the reaction was stopped by the addition of 100 μl of Malachite green solution (Biomol green, Enzo Life Sciences), and the released phosphate was quantified by measurement of the absorbance at 620 nm.

### *E*. *coli* functional complementation

The *E*. *coli* thermosensitive strains BWTs*bacA* and BWPGPTs, carrying multiple chromosomal gene deletions and harboring a *bacA*- or *pgpB*-expressing plasmid, respectively, whose replication is impaired at 42°C, have been previously described [7]. These strains were transformed by the p*Trc*His30-based plasmids encoding the different *H*. *pylori* PAP2 and PgpA encoding genes. Isolated transformants were subcultured at 30°C in liquid 2YT medium supplemented with 100 μg/ml ampicillin and, when the absorbance of the culture reached 0.5, the cell suspension was diluted to 10^−5^ in 2YT medium and 100 μl aliquots were plated onto two ampicillin-containing 2YT agar plates which were incubated at either 30°C or 42°C for 24 h. The colony forming units (CFU) were numerated on each plate and the functional complementation of the thermosensitive mutants was evaluated by the capacity of the transformants to equally grow at both temperatures.

### Transformation rate

Precultures of *H*. *pylori* were started from glycerol stocks routinely stored at -80°C, plated onto 10% horse blood agar medium and incubated at 37°C for 24 h in a microaerobic atmosphere (6% O_2_, 10% CO_2_, 84% N_2_). Amplification of the pre-culture was performed in the same conditions such as pre-cultures in new plates at 37°C for 24 h in a microaerobic atmosphere. 50 μl of suspension in BHI at OD_600nm_ = 20 were made until the amplification plate and mixed with 10 ng of Topo TA*ΔlpxE* plasmid. The whole mixture was put on a non-selective plate onto 10% horse blood agar medium and incubated at 37°C for 24 h in a microaerobic atmosphere. The transformation spot was then diluted (10^−1^ to 10^−8^). 50 μl of the non-diluted suspension and 10 μl of the 10^−1^ to 10^−4^ dilutions were spread on 10% horse blood agar medium supplemented with chloramphenicol (4 μg/ml), kanamycin (20 μg/ml) and gentamycin (5 μg/ml) ± IPTG 1mM to estimate the number of transformants. The 10^−5^ to 10^−8^ dilutions were spread on non-selective plates containing 10% horse blood agar medium supplemented with chloramphenicol (4 μg/ml), kanamycin (20 μg/ml) ± IPTG 1mM to estimate the total number of bacteria. The CFU of all plates incubated at 37°C for 5 days in a microaerobic atmosphere were enumerated. The transformation rate was expressed by the number of transformants/total cfu/μg of DNA TopoTA*ΔlpxE*.

### Determination of Minimum Inhibitory Concentration (MIC) of polymyxin B

Strains were grown routinely on amplification plates. Two ml of suspension in 0.9% NaCl at OD_600nm_ = 0.75 were made from the amplification plates and spread by inundation onto Mueller Hinton medium (Difco) supplemented with 10% of FBS and 2,3,5-triphenyltetrazolium chloride (TTC, Sigma) (40 μg/ml). Polymyxin B was diluted by serial dilutions of two (16384 μg/ml down to 0.5 μg/ml). Once the square plates with bacterial lawns were dried, 10 μl of each dilution of polymyxin B was dropped on and allowed to dry. All plates were then incubated at 37°C for 3 days in a microaerobic atmosphere. TTC is a non-toxic dye which colors in red the alive bacteria. MICs were determined as the lowest concentration of polymyxin B leading to a clear halo of inhibition.

### Determination of Minimum Inhibitory Concentration (MIC) by Etest

Strains were grown routinely on amplification plates. Two ml of suspension in 0.9% NaCl at OD_600nm_ = 0.75 were made from the amplification plates and spread by inundation onto 10% horse blood agar medium. Once the square plates with bacterial lawns were dried, Etes, Biomérieux, with different antibiotics targeting the cell wall (Vancomycin, Teicoplanin, Daptomycin and Colistin) were put on plate and incubated at 37°C for 48 h in a microaerobic atmosphere. MICs were determined as the lowest concentration of antibiotics leading to a clear halo of inhibition.

### Colonization

OF1 female mice purchased from Charles River Laboratories aged 5 weeks were infected by gavage with feeding needles with X47 strain (2 × 10^8^ bacteria per mouse). Colonization rates were determined after 1, 4, 7, 15 and 32 days by enumeration of CFU per gram of stomach. Mice were euthanized with CO_2_ and the stomachs were ground and homogenized in peptone broth. The samples were then diluted and spread on blood agar plates supplemented with 10 μg/ml of nalidixic acid, to inhibit the growth of resident bacteria from the mouse forestomach and 20 μg/ml of kanamycin for *hupA* mutant strain. The CFU were enumerated after 5 days of incubation under microaerobic conditions. The results of two independent colonization experiments (seven mice by cage) were pooled and a one tailed Mann-Whitney test was used to determine statistical significance of observed differences (GraphPad Prism v5.0 GraphPad Software, CA).

### Isolation of lipid A for mass spectrometry analysis

For isolation of lipid A, *H*. *pylori* cultures were grown to an OD_600_ of ~0.6. Lipid A chemical extraction was carried out after mild acidic hydrolysis of LPS as previously described [32,33]. For visualization of lipid A by mass spectrometry, lipids were analyzed using MALDI-TOF (ABI 4700 Proteomic Analyzer) in the negative-ion linear mode similar to previously described [34,35]. Briefly, lipid A samples were dissolved in a mixture of chloroform-methanol (4:1, vol/vol), with 1 μL of sample mixed with 1 μL of matrix solution consisting of 5-chloro-2meracaptobenzothiazole (CBMT) (20 mg/mL) resuspended in chloroform-methanol-water (4:4:1, vol/vol/vol) mixed with saturated ammonium citrate (20:1, vol/vol), and 1 μL of sample-matrix mixture was loaded on to MALDI target plate.

## Supporting information

S1 Fig Purification and characterization of PgpA from *H*. *pylori*. (A) SDS-PAGE analysis of His_6_-PgpA purification. Coloration was performed with Coomassie Blue R-250. Purified PgpA protein is indicated with an asterisk in the elution fraction. (B) Mg^2+^-dependence of PgpA PGPase activity. The results are expressed as the percentage of the optimal activity found at a final concentration of 6 mM of MgCl_2_. The observed molecular weight of recombinant PgpA was lower than the calculated one (16,9 kDa).
(TIF)

S2 Fig Membrane phospholipids of *Helicobacter pylori*. TLC analysis of total lipid extracts from N6 (1) WT strain and the four single mutants (2) *lpxE*∷Gm; (3) *hp0350*∷Km; (4) *lpxF*∷Km; (5) *hupA*∷Km grown to exponential phase in BHI medium. Left panel contains control phospholipids: CL: Cardiolipin; PC: Phosphotidylcholine; PE: Phosphotidylethanolamine; PG: Phosphotidylglycerol; PS: Phosphotidylserine; Lip. EC: Lipid extracts from *E*. *coli*.
(TIF)

S1 Table Oligonucleotides used in this study. Oligonucleotides which have an underlined nucleotide sequence, the sequence corresponds to the restriction enzyme cutting site.
(DOCX)
