# Supplementary figures and images for: HupA, the main undecaprenyl pyrophosphate and phosphatidylglycerol phosphate phosphatase in Helicobacter pylori is essential for colonization of the stomach
Source: PLoS Pathog. 2019 Sep 5;15(9):e1007972. doi: 10.1371/journal.ppat.1007972 (PMC6748449; doi:10.1371/journal.ppat.1007972)

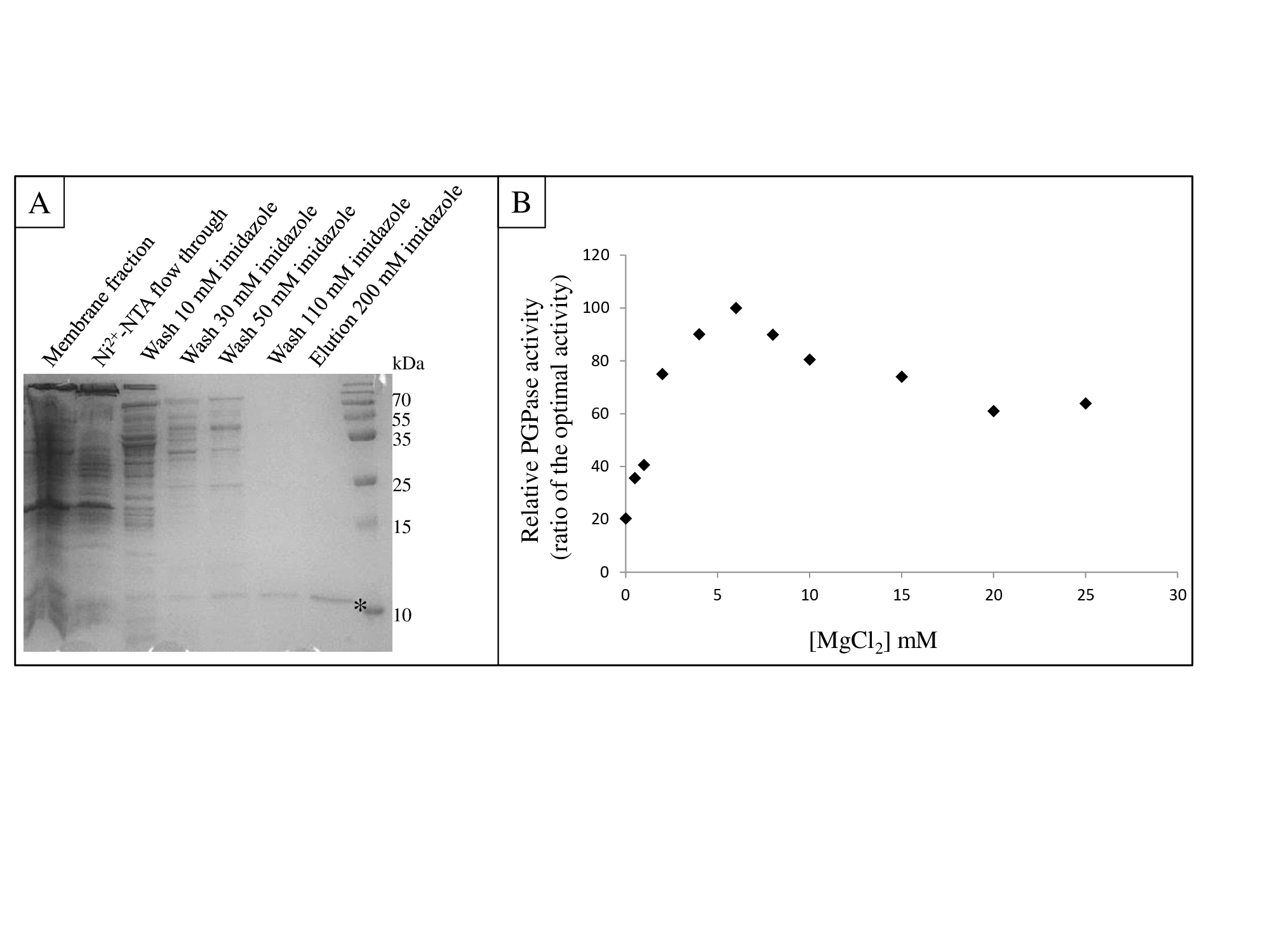

Supplement: S1 Fig — (A) SDS-PAGE analysis of His6-PgpA purification. Coloration was performed with Coomassie Blue R-250. Purified PgpA protein is indicated with an asterisk in the elution fraction. (B) Mg2+-dependence of PgpA PGPase activity. The results are expressed as the percentage of the optimal activity found at a final concentration of 6 mM of MgCl2. The observed molecular weight of recombinant PgpA was lower than the calculated one (16,9 kDa). (TIF) [file ppat.1007972.s001.tif]

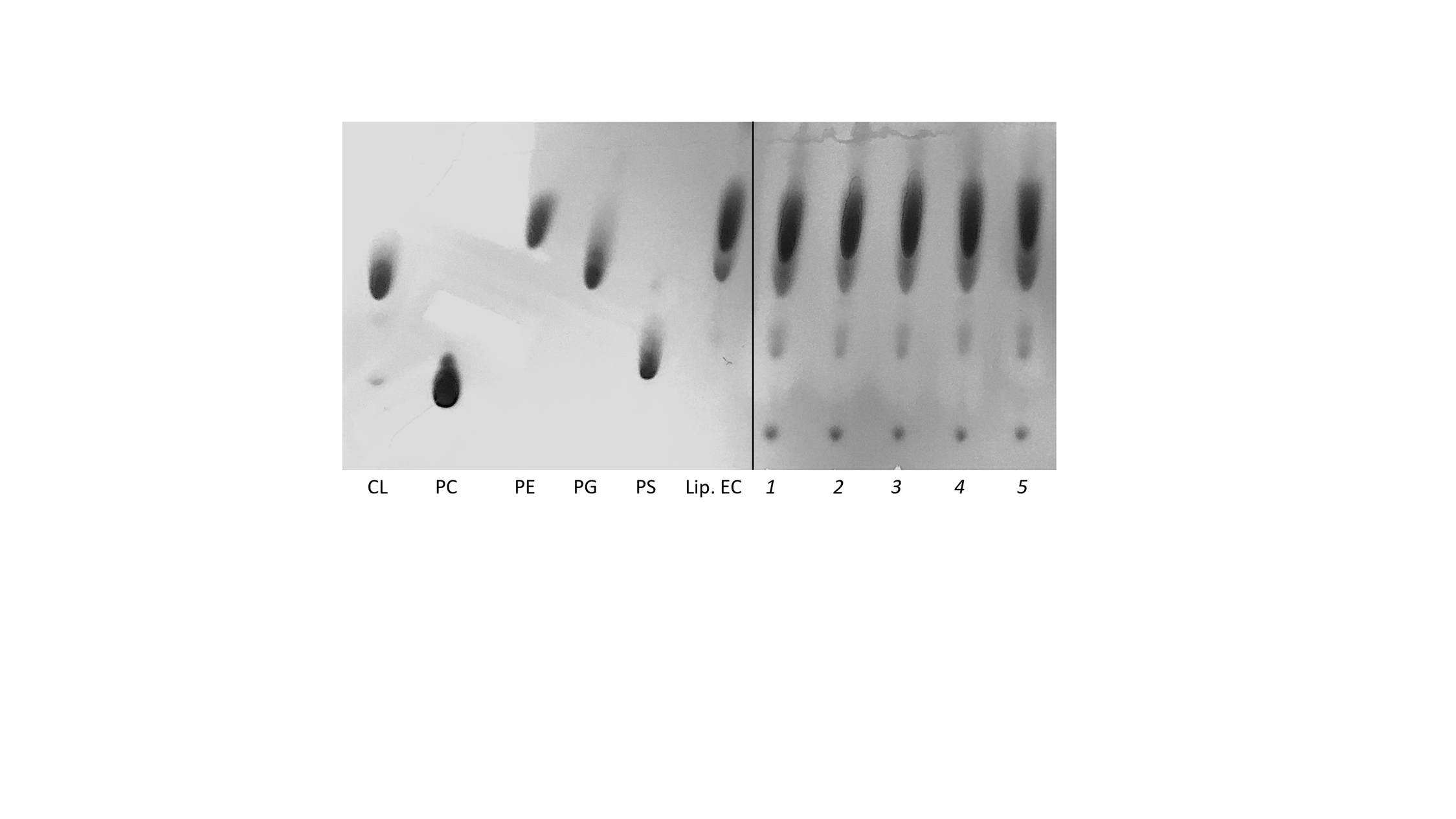

Supplement: S2 Fig — TLC analysis of total lipid extracts from N6 (1) WT strain and the four single mutants (2) lpxE∷Gm; (3) hp0350∷Km; (4) lpxF∷Km; (5) hupA∷Km grown to exponential phase in BHI medium. Left panel contains control phospholipids: CL: Cardiolipin; PC: Phosphotidylcholine; PE: Phosphotidylethanolamine; PG: Phosphotidylglycerol; PS: Phosphotidylserine; Lip. EC: Lipid extracts from E. coli. (TIF) [file ppat.1007972.s002.tif]
