# Supplementary material for: HupA, the main undecaprenyl pyrophosphate and phosphatidylglycerol phosphate phosphatase in Helicobacter pylori is essential for colonization of the stomach
Source: PLoS Pathog. 2019 Sep 5;15(9):e1007972. doi: 10.1371/journal.ppat.1007972 (PMC6748449; doi:10.1371/journal.ppat.1007972)
Supplement: S1 Table — Oligonucleotides which have an underlined nucleotide sequence, the sequence corresponds to the restriction enzyme cutting site. (DOCX) [file ppat.1007972.s003.docx]

**S1 Table**. **Oligonucleotides used in this study.**
